# Extracorporeal membrane oxygenation and iliac vein injury detected with bedside ultrasound

**DOI:** 10.1186/2036-7902-6-S1-A4

**Published:** 2014-01-31

**Authors:** Sang Ook Ha, Jae Seok Park, So Hee Park

**Affiliations:** 1 Department of Emergency Medicine, Asan Medical Center, College of Medicine, University of Ulsan, Seoul, Korea; 2 Department of Pulmonary and Critical care Medicine, Asan Medical Center, College of Medicine, University of Ulsan, Seoul, Korea

## 

The use of extracorporeal membrane oxygenation (ECMO) has increased after 2009 pandemic H1N1 infection, and the ECMO related complications have also increased. Especially mechanical vessel injury due to catheter cannulation seems less frequency than other complications, but there is a risk of hemorrhagic shock requiring caution. We experienced a case of early detection of iliac vein injury by bedside ultrasound and successful management with graft-stent during ECMO operation. A 56-years-old female patient with non small cell lung cancer developed endobronchial obstruction, and ECMO was applied for the ECMO-assisted rigid bronchoscopy. During catheter cannulation, hypovolemic shock was developed due to right external iliac vein injury. We early detected the hemorrhage with bedside ultrasound, the hemorrhage was effectively managed with graft stent on ECMO.

**Figure 1:**
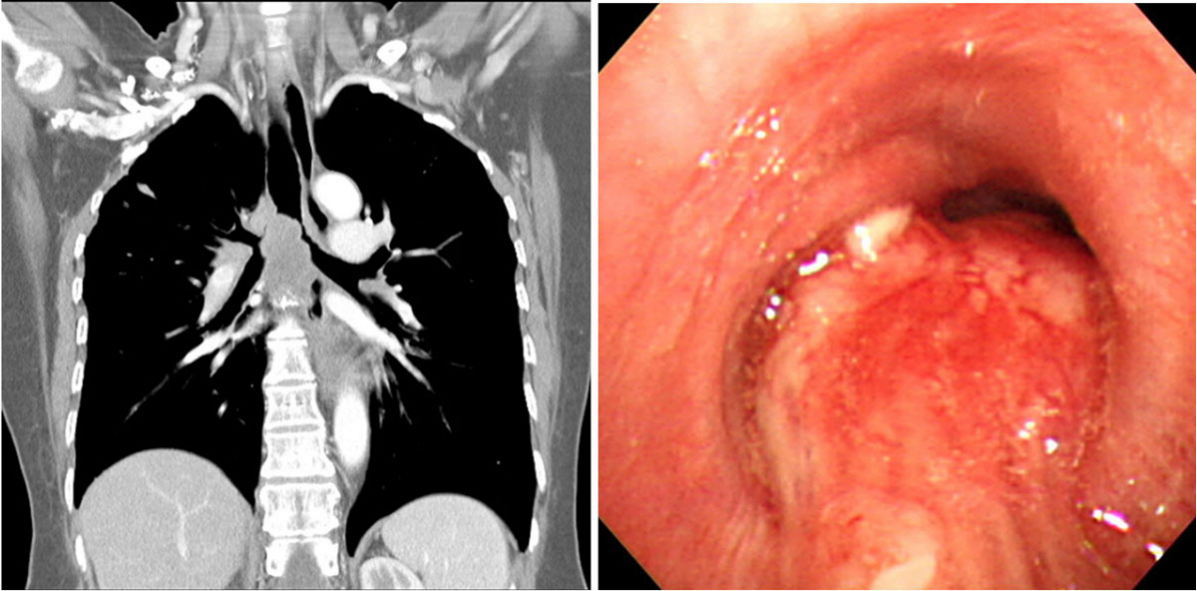
The chest CT and fiberoptic bronchoscopy show mass in carina and right main bronchus.

**Figure 2:**
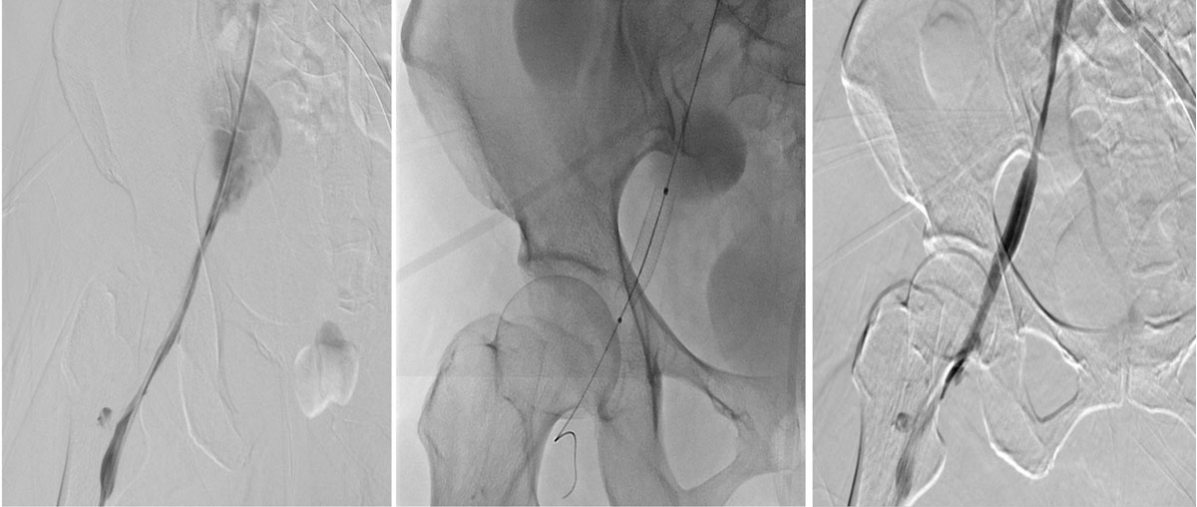
Angiogram demonstrate hemorrhage of right external iliac vein after catheter cannulation for ECMO (a) and graft stent is successfully inserted. (b, c)

